# Validation of a *Stenotrophomonas maltophilia* bloodstream infection prediction score in the hematologic malignancy population

**DOI:** 10.1007/s00277-024-05686-z

**Published:** 2024-03-08

**Authors:** Emily L. Gill, Christian M. Gill, Colleen McEvoy

**Affiliations:** 1 Department of Pharmacy, Barnes Jewish Hospital, 216 S. Kingshighway Blvd, Mailstop 90-52-41, Saint Louis, MO 63110 USA; 2 Department of Pharmacy, SSM-Health St. Louis University Hospital, Saint Louis, MO USA; 3 Center for Anti-Infective Research and Development, Hartford Hospital, Hartford, CT USA; 4 Division of Pulmonary and Critical Care, Washington University School of Medicine, Saint Louis, MO USA

**Keywords:** *Stenotrophomonas maltophilia*, Bloodstream infection, Hematologic malignancy, Risk score

## Abstract

*Stenotrophomonas maltophilia* (SM) bloodstream infections (BSIs) contribute to significant mortality in hematologic malignancy (HM) and hematopoietic stem cell transplantation (HSCT) patients. A risk score to predict SM BSI could reduce time to appropriate antimicrobial therapy (TTAT) and improve patient outcomes. A single center cohort study of hospitalized adults with HM/HSCT was conducted. Patients had ≥ 1 blood culture with a Gram-negative (GN) organism. A StenoSCORE was calculated for each patient. The StenoSCORE2 was developed using risk factors for SM BSI identified via logistic regression. Receiver operating characteristic (ROC) curves were plotted. Sensitivity and specificity for the StenoSCORE and StenoSCORE2 were calculated. Thirty-six SM patients and 534 non-SM patients were assessed. A StenoSCORE ≥ 33 points was 80% sensitive, 68% specific, and accurately classified 69% of GN BSIs. StenoSCORE2 variables included acute leukemia, prolonged neutropenia, mucositis, ICU admission, recent meropenem and/or cefepime exposure. The StenoSCORE2 performed better than the StenoSCORE (ROC AUC 0.84 vs. 0.77). A StenoSCORE2 ≥ 4 points was 86% sensitive, 76% specific, and accurately classified 77% of GN BSIs. TTAT was significantly longer for patients with SM BSI compared with non-SM BSI (45.16 h vs. 0.57 h; *p* < 0.0001). In-hospital and 28-day mortality were significantly higher for patients with SM BSI compared to non-SM BSI (58.3% vs. 18.5% and 66.7% vs. 26.4%; p-value < 0.0001). The StenoSCORE and StenoSCORE2 performed well in predicting SM BSIs in patients with HM/HSCT and GN BSI. Clinical studies evaluating whether StenoSCORE and/or StenoSCORE2 implementation improves TTAT and clinical outcomes are warranted.

**Supplementary Information:**

The online version contains supplementary material available at 10.1007/s00277-024-05686-z.

## Introduction

*Stenotrophomonas maltophilia* is a multidrug resistant, Gram-negative bacteria that has emerged as an opportunistic and life-threatening pathogen in immunosuppressed and critically ill patients [1, 2]. *S. maltophilia* bloodstream infections (BSI) are associated with high mortality rates ranging from 21 to 69% [3]. Patients with hematologic malignancy and hematopoietic stem cell transplant (HSCT) are at an increased risk for developing *S. maltophilia* BSI due to widespread use of central venous catheters (CVC), neutropenia, exposure to broad-spectrum antibiotic therapy, and prolonged hospital lengths of stay [1, 3]. Prompt initiation of treatment active against *S. maltophilia* poses a clinical challenge due to intrinsic resistance to commonly employed antimicrobials for Gram-negative infections, including most β-lactams and aminoglycosides [4]. Historically, trimethoprim-sulfamethoxazole, minocycline, and/or levofloxacin have been first-line treatment options for *S. maltophilia* infections, however; these agents are not commonly initiated as empiric therapy for Gram-negative BSIs due to the low overall prevalence of *S. maltophilia* and toxicities associated with their use (e.g., trimethoprim-sulfamethoxazole) [2, 4].

Currently available rapid molecular diagnostic tests have the capabilities to quickly identify *S. maltophilia* from blood cultures within hours, however, many healthcare institutions do not have access to this technology [5]. Among patients with hematologic malignancy/HSCT and Gram-negative BSI, the use of a risk score to identify patients with an increased likelihood of having *S. maltophilia* BSI could reduce time to appropriate antimicrobial therapy and thus reduce mortality. The StenoSCORE was developed in an observational cohort of patients with hematologic malignancy/HSCT and Gram-negative BSI to better identify patients at risk for *S. maltophilia* BSI [4]. The scoring tool utilizes five variables which have either previously been identified as risk factors for *S. maltophilia* BSI or were found to be significant risk factors within the cohort. Possible scores range from 0 to 77 points with scores ≥ 41 points correctly classifying > 80% of Gram-negative BSI observations as *S. maltophilia* infections within the study [4]. Although the StenoSCORE represents a pragmatic tool for identifying patients that may benefit from *S. maltophilia* active therapy in the setting of Gram-negative BSI, it has not been externally validated outside of the original study. We sought to determine the utility of the StenoSCORE in a cohort of hematologic malignancy/HSCT patients with documented Gram-negative BSI as well as to determine if alternative variables better predicted *S. maltophilia* BSI within this patient population.

## Methods

### Study design and patient population

This was a single center, cohort study of adult patients with hematologic malignancy/HSCT admitted to Barnes Jewish Hospital between 5/1/2018 and 2/28/2023. Patients aged ≥ 18 years with hematologic malignancy actively receiving chemotherapy and/or received a HSCT within the preceding 12 months and had at least one blood culture positive for Enterobacterales or non-fermenting Gram-negative bacilli within the study period were included. A cohort of patients with *S. maltophilia* BSI was identified to serve as the primary case population of interest. For comparison, a second cohort of patients with BSI due to non-*S. maltophilia* Gram-negative organisms was subsequently identified to serve as controls. Only unique hospital encounters for BSIs were included in the study and patients could only be included into one of the two cohorts. Only the first episode of BSI meeting criteria was included for patients with multiple BSIs within the same hospital encounter.

### Data collection and definitions

Patients were identified for study inclusion using ICD10 codes for hematologic malignancy, HSCT, and Gram-negative bacteremia. Relevant patient characteristics collected at the time of index culture included: malignancy diagnosis, comorbid conditions, vital signs, absolute neutrophil count (ANC), presence of mucositis, and indwelling CVC. Hospital and intensive care unit (ICU) admission and discharge data for both the current encounter and all hospital encounters in the preceding 3 months of index culture were collected. All microbiologic data in the 6 months preceding the index culture, antimicrobial therapy prescribed in the previous 3 months, and antimicrobial therapy for current BSI were reviewed.

BSI was defined as at least one blood culture positive for an included Gram-negative organism. Active chemotherapy was defined as receipt of a parenteral chemotherapeutic agent within the month preceding the index culture. Presence of mucositis was ascertained via chart review for oral ulcers noted within the patient’s active problem list or on physical exam. Consistent with the previous publication, neutropenia was defined as an ANC < 1500 cells/mm^3^ [4]. Appropriate antimicrobial therapy was defined as receipt of an appropriately dosed antimicrobial deemed susceptible based on the index culture antimicrobial susceptibility report determined using disk diffusion per CLSI standards [6]. An isolate was considered multidrug resistant if the organism was carbapenem resistant, the organism was an extended-spectrum β-lactamase producer, or if the culture contained *Pseudomonas aeruginosa*, *Acinetobacter baumannii*, *Achromobacter species*, or Enterobacterales that was resistant to at least one drug in three or more of the following antibiotic categories: piperacillin-tazobactam, extended-spectrum cephalosporins, fluoroquinolones, aminoglycosides, or carbapenems [7]. If *A. baumannii* was cultured, the isolate was considered multidrug resistant if it was resistant to ampicillin-sulbactam [7]. An isolate was considered a carbapenem resistant organism if it was resistant to at least one carbapenem or produced a carbapenemase as determined by use of the Xpert Carba-R assay (Cepheid, Sunnyvale, CA, USA) [8].

### Statistical analysis

The primary endpoints of interest were identification of variables associated with *S. maltophilia* BSI as compared with non-*S. maltophilia* Gram-negative BSI. Categorical data were compared using Chi-square or Fisher’s exact test as appropriate. Continuous data were compared using Mann-Whitney U test. To assess independent risk factors for in-hospital mortality, a post-hoc multivariable logistic regression was performed using variables with a p-value of < 0.05 on univariable analysis.

A StenoSCORE was calculated for each patient using predetermined variables and point assignments described in the previously mentioned study (Table 1) [4]. The receiver operating characteristic (ROC) curve was plotted and area under the curve (AUC) was calculated. Discrimination was evaluated using ROC AUC with its corresponding 95% confidence interval and calibration was assessed using the Hosmer-Lemeshow goodness-of-fit test. Sensitivity, specificity, positive predictive value, and negative predictive value of the StenoSCORE were calculated to evaluate for an optimal point cutoff for *S. maltophilia* BSI prediction.

**Table 1:** Comparison of StenoSCORE and StenoSCORE2

StenoSCORE		StenoSCORE2	
Variable	Point Assignment	Variable	Point Assignment
**Acute Leukemia**	1	**Acute Leukemia**	1
**Mucositis**	23	**Mucositis**	1
**CVC**	22	**Neutropenia ≥ 7 days**	2
**Carbapenem ≥ 3 days**	19	**ICU on index culture**	1
**ANC** ** 0–99** ** 100–499** ** 500–999** ** 1000–1499** ** ≥1500**	0 3 6 9 12	**Carbapenem ≥ 3 days**	2
		**Cefepime ≥ 3 days**	1
**Total Points**	**77**	**Total Points**	**8**

To evaluate variables associated with *S. maltophilia* BSI in our patient population, univariable analysis was used to assess the unadjusted association between independent variables and *S. maltophilia* BSI. Using a significance level of < 0.05, six variables were selected for inclusion into the binary logistic regression which would then comprise the StenoSCORE2. Points assigned for each variable in the risk score were derived from scaling variables by the smallest model coefficient in the binary logistic regression and rounding the result to the nearest integer. The total StenoSCORE2 was calculated by summation of the points associated with each variable applicable to a given patient. The model was assessed by plotting the ROC curve and determining discrimination and calibration as previously described [4]. Sensitivity, specificity, positive predictive value, and negative predictive value of the StenoSCORE2 were calculated. Statistical analyses were performed using SPSS Statistics version 25 (IBM corp, Armonk, NY, USA).

## Results

In total, 36 patients with *S. maltophilia* BSI and 534 patients with non-*S. maltophilia* Gram-negative BSI were identified for inclusion. Organisms included in the non-*S. maltophilia* Gram-negative BSI group are listed in Table S1. Baseline characteristics for the study population are listed in Table 2. Compared with non-*S. maltophilia* Gram-negative BSI patients, patients with *S. maltophilia* BSI were more likely to have acute myeloblastic or lymphoblastic leukemia (75% vs. 46.8%; p-value < 0.0001), experience neutropenia for ≥ 7 days prior to the index culture date (77.8% vs. 35.2%; p-value < 0.0001), have mucositis (36.1% vs. 13.1%; p-value < 0.0001), ICU admission within 12 h of index culture (44.4% vs. 24.5%; p-value 0.008), have a prior history of *S. maltophilia* colonization or infection in the preceding 6 months (13.9% vs. 0.2%; p-value < 0.0001), and have received ≥ 3 days of carbapenem therapy (63.9% vs. 17.4%; p-value < 0.0001), cefepime (63.9% vs. 27.3%; p-value < 0.0001), or piperacillin-tazobactam (8.3% vs. 1.3%; p-value < 0.0001) within the preceding 3 months. The time from hospitalization to index blood culture was significantly longer in the *S. maltophilia* group compared with the non-*S. maltophilia* Gram-negative BSI group (19 days vs. 10 days; p-value < 0.0001). Degree of neutropenia and presence of CVC did not differ between groups. Time from index culture to initiation of appropriate antimicrobial therapy was 0.57 and 45.16 h in the non-*S. maltophilia* BSI and *S. maltophilia* BSI groups, respectively (p-value < 0.0001). Both in-hospital and 28-day mortality were significantly higher in the *S. maltophilia* BSI group compared with the non-*S. maltophilia* BSI group (58.3% vs. 18.5%, p-value < 0.0001 and 66.7% vs. 26.4%; p-value < 0.0001). In an exploratory multivariable analysis, *S. maltophilia* BSI and time to appropriate antimicrobials greater than 60 min were both independent predictors of in-hospital mortality (Table 3).

**Table 2:** Baseline Characteristics

	Non-*S. maltophilia* (*n* = 534)	*S. maltophilia* (*n* = 36)	P value
Age, median (IQR)	64 (56, 70)	60 (52, 69)	---
Gender, Male, N (%)	301 (56.4)	21 (58.3)	---
Underlying malignancy, N (%)			
Acute myeloblastic or lymphoblastic leukemia	250 (46.8)	27 (75.0)	0.001
Lymphoma^a^	35 (6.6)	3 (8.3)	---
Multiple myeloma	110 (20.5)	0 (0)	0.002
Myelodysplastic syndrome	34 (6.4)	4 (11.1)	---
Other^b^	105 (19.7)	2 (5.6)	0.036
HSCT within preceding 12 months, N (%)	223 (41.8)	15 (41.7)	---
Neutropenic, N (%)	454 (85)	31 (86.1)	---
Absolute neutrophil count, N (%)			---
Severe: 0-499	419 (78.5)	30 (83.3)	
Moderate: 500–999	24 (4.5)	1 (2.8)	
Mild: 1000–1499	11 (2.1)	0 (0)	
Normal: ≥ 1500	80 (15)	5 (13.9)	
Neutropenia ≥ 7 days, N (%)	188 (35.2)	28 (77.8)	< 0.0001
Mucositis, N (%)	70 (13.1)	13 (36.1)	< 0.0001
Central venous catheter, N (%)	484 (90.6)	36 (100)	---
Severity of Illness			
Charlson comorbidity index, median (IQR)	5 (4, 8)	5 (3, 7)	---
Pitt bacteremia score, median (IQR)	1 (0, 2)	2 (0, 4)	---
ICU admission within 12 h of index culture, N (%)	131 (24.5)	16 (44.4)	0.008
Time from hospital admission to index culture, days, median (IQR)	10 (0, 17)	19 (10, 24)	< 0.0001
Time from index culture to appropriate antibiotic, hours, median (IQR)^c^	0.57 (0.15, 2.19)	45.16 (16.73, 64.78)	< 0.0001
Prior MDR Gram-negative colonization/infection in preceding 6 months N (%)	6 (1.1)	1 (2.8)	---
Prior *S. maltophilia* colonization/infection in preceding 6 months, N (%)	1 (0.2)	5 (13.9)	< 0.0001
Antibiotic exposure in preceding 3 months, N (%)			
Carbapenem ≥ 3 days	93 (17.4)	23 (63.9)	< 0.0001
Cefepime ≥ 3 days	146 (27.3)	23 (63.9)	< 0.0001
Piperacillin-tazobactam ≥ 3 days	7 (1.3)	3 (8.3)	< 0.02
Prophylactic fluoroquinolone	137 (25.7)	21 (58.3)	< 0.0001
Prophylactic oral beta-lactam	36 (6.7)	7 (19.4)	0.013
Hospitalization in the preceding 3 months, N (%)	241 (45.1)	14 (38.9)	---
Days in hospital, median (IQR)	14 (6, 29)	24 (15, 29)	---
In-hospital mortality, N (%)	99 (18.5)	21 (58.3)	< 0.0001
28-day mortality, N (%)	141 (26.4)	24 (66.7)	< 0.0001
StenoSCORE, median (IQR)	23 (22, 41)	42 (34, 64)	< 0.0001
StenoSCORE2, median (IQR)	1 (0, 3)	5 (4, 6)	< 0.0001

^a^Includes T-cell lymphoma, anaplastic large cell lymphoma, angioimmunoblastic T-cell lymphoma, NK-cell lymphoma, Burkitt lymphoma, cutaneous T-cell lymphoma, marginal Zone B-cell lymphoma, follicular lymphoma, Hodgkin lymphoma, lymphoblastic lymphoma, mantle cell lymphoma, non-Hodgkin lymphoma, peripheral T-cell lymphoma, B-cell lymphoma

^b^Includes acute monoblastic/monocytic leukemia, acute myelomonocytic leukemia, acute promyelocytic leukemia, aplastic anemia, chronic lymphocytic leukemia, chronic myeloid leukemia, chronic myelomonocytic leukemia, monoclonal gammopathy, myelofibrosis, prolymphocytic leukemia of B-cell type, prolymphocytic leukemia of T-cell type

^c^Antimicrobial susceptibility testing data were not available for 21 patients (20 patients in the non-*S. maltophilia* group and 1 patient in the *S. maltophilia* group), therefore, those patients were excluded from analysis of this outcome

**Table 3:** Factors Associated with In-Hospital Mortality using Multivariable Regression

Variable	Odds ratio (95% CI)
Acute myeloid leukemia or acute lymphoblastic leukemia	0.75 (0.46–1.25)
Neutropenia ≥ 7 days	0.85 (0.52–1.38)
Appropriate antimicrobial administration within 60 min	3.25 (1.64–6.44)
*S. maltophilia* bacteremia	4.89 (2.38–10.06)

**Fig. 1:**
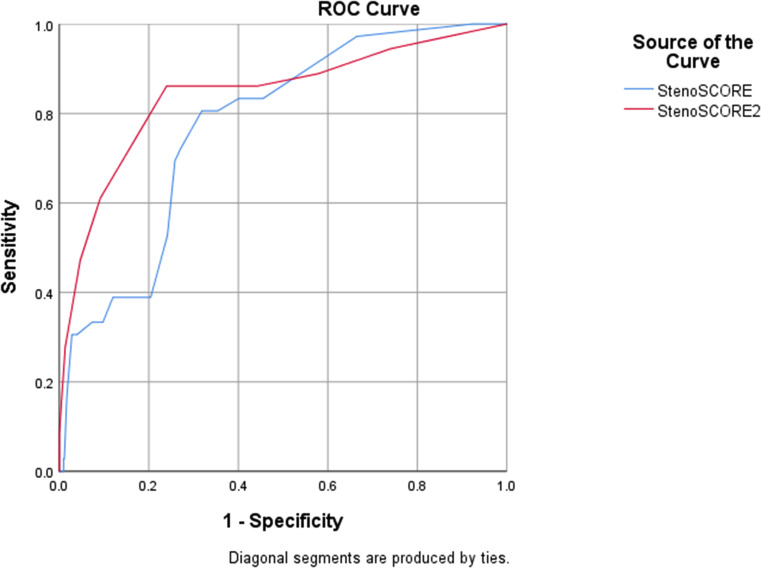
Receiver operator characteristic curves illustrating the sensitivity and specificity of the StenoSCORE and StenoSCORE2. The StenoSCORE exhibited acceptable discrimination with an AUC of 0.77 (95% CI 0.70-0.84) while the StenoSCORE2 exhibited excellent discrimination with an AUC of 0.84 (95% CI 0.76-0.92)

When assessing StenoSCOREs between groups, patients with *S. maltophilia* BSI had significantly higher scores compared with patients with non-*S. maltophilia* BSI (42 vs. 23; p-value < 0.0001). ROC curve analysis of the StenoSCORE performance in our cohort produced an AUC 0.77 (95% CI 0.70–0.84) indicating acceptable discrimination (Fig. 1). Table 4 summarizes the sensitivity, specificity, and accuracy of the StenoSCORE at various point cutoffs. A StenoSCORE ≥ 33 had the highest combined sensitivity and specificity with values of 80% and 68%, respectively.

**Table 4:** Sensitivity, Specificity, and Accuracy of StenoSCORE and StenoSCORE2

StenoSCORE				StenoSCORE2			
Risk Score Cutoff Point	Sensitivity (%)	Specificity (%)	Accurately Classified (%)	Risk Score Cutoff Point	Sensitivity (%)	Specificity (%)	Accurately Classified (%)
**≥ 30**	80	67	68	**≥ 3**	86	56	58
**≥ 33**	80	68	69	**≥ 4**	86	76	77
**≥ 38**	69	74	74	**≥ 5**	61	91	89

After assessing the performance of the StenoSCORE in our cohort, we then sought to determine if a different combination of variables within our cohort would better predict *S. maltophilia* BSI. Based on the results of univariable analysis, acute leukemia (acute myeloid or acute lymphoblastic leukemia), neutropenia ≥ 7 days, mucositis, ICU admission within 12 h of index culture, prior meropenem exposure ≥ 3 days, and prior cefepime exposure ≥ 3 days were selected for inclusion in the binary logistic regression model (Table S2). Neutropenia ≥ 7 days [OR 4.10 (95% CI 1.67–10.08)], mucositis [OR 2.51 (95% CI 1.08–5.79)], meropenem exposure [OR 4.63 (2.09–10.28)], and cefepime exposure [OR 2.55 (95% CI 1.14–5.69)] remained significant predictors of *S. maltophilia* BSI in the regression model. The model demonstrated good calibration with a Hosmer and Lemeshow chi-square 13.4, p-value = 0.06. Multicollinearity was not detected within the model as evidenced by variance inflation factors < 1.3 in regression analysis. Risk score points for the StenoSCORE2 are summarized in Table 1. Possible StenoSCORE2 values ranged from 0 to 8. The median StenoSCORE2 was 5 in the *S. maltophilia* BSI group compared with 1 in the non-*S. maltophilia* BSI group (p- value < 0.0001). ROC curve analysis of the StenoSCORE2 performance in our cohort produced an AUC 0.84 (95% CI 0.76–0.92) indicating excellent discrimination (Figure 1). Table 4 summarizes the sensitivity, specificity, and accuracy of the StenoSCORE2 at various point cutoffs. A StenoSCORE2 ≥ 4 had a sensitivity of 86%, specificity of 76%, and accurately identified 77% of *S. maltophilia* BSIs.

## Discussion

In our cohort of patients with hematologic malignancy and/or HSCT, *S. maltophilia* BSI accounted for only 6.3% of Gram-negative BSI but was associated with significant in-hospital mortality. With in-hospital mortality rates of 58.3% seen in patients with *S. maltophilia* BSI compared with 18.5% in patients the non-*S. maltophilia*, our findings highlight the clinical importance of *S. maltophilia* BSI as a contributor to poor outcomes in this patient population. Recognition of risk factors for *S. maltophilia* BSI and prompt initiation of antimicrobial therapy active against *S. maltophilia* will be important to help close this wide mortality gap and improve outcomes in this vulnerable patient population.

Numerous studies have evaluated risk factors for *S. maltophilia* infection and BSI in patients with hematologic malignancy and/or HSCT [4, 9–18]. Our study was the first to systematically apply a collection of predetermined risk factors for *S. maltophilia* BSI known as the StenoSCORE and validate its utility in a second cohort of patients with hematologic malignancy and/or HSCT and Gram-negative BSI. To date, studies evaluating risk factors for *S. maltophilia* BSI have largely been single center or localized to a single health system [4, 9–18]. Regional differences in patient populations, oncologic treatment practices, and changes in treatment practices over time have led to heterogeneity among reported risk factors and outcomes for *S. maltophilia* BSI in patients with hematologic malignancy and/or HSCT. Consistent with the previous StenoSCORE publication, we identified characteristics which differentiate *S. maltophilia* BSI from non-*S. maltophilia* BSI in all patients with hematologic malignancy undergoing active chemotherapy and/or recent HSCT [4]. In contrast to another published risk scoring tool which aims to differentiate between *S. maltophilia* BSI and *P. aeruginosa* BSI in patients with hematologic malignancy, both the StenoSCORE and StenoSCORE2 can differentiate *S. maltophilia* BSI from BSI with Enterobacterales or other non-fermenters [12]. This is notable as the StenoSCORE and StenoSCORE2 may have greater generalizability given the greater prevalence of BSI caused by Enterobacterales as opposed to *P. aeruginosa* in this patient population as determined by the present study (Table S1) and others [19]. In our study, patients with *S. maltophilia* BSI had a median StenoSCORE of 42 which corroborated the previous report that StenoSCOREs ≥ 41 were able to discriminate *S. maltophilia* BSI from non-*S. maltophilia* BSI [4]. Additional multicenter students are warranted to confirm this finding.

Commonly cited risk factors for *S. maltophilia* BSI include carbapenem exposure, prolonged hospital stay, admission to the ICU, severe neutropenia, indwelling CVC, mucositis, and previous *S. maltophilia* colonization [4, 10–12, 14, 16–18]. Within our cohort, ANC at the time of index blood culture did not differ between patients with *S. maltophilia* BSI and non-*S. maltophilia* BSI. Additionally, the high proportion of patients within our cohort with an indwelling CVC at the time of index culture diminished the utility of evaluating CVC as a risk factor for *S. maltophilia* BSI. Thus, the resultant StenoSCORE2 derived from our cohort maintained acute leukemia, mucositis, and carbapenem exposure as important risk factors for *S. maltophilia* BSI but removed CVC and ANC as contributing risk variables (Table 1). Consistent with previous analyses, prolonged neutropenia and cefepime exposure were independent predictors of *S. maltophilia* BSI in multivariable analysis [10, 12, 17]. Additional variables found to be significantly associated with *S. maltophilia* BSI in univariable analysis yet not included in the multivariable analysis were increased time from hospital admission to culture, previous colonization or infection with *S. maltophilia*, piperacillin-tazobactam exposure, and use of antimicrobial prophylaxis. These variables were not selected for inclusion in the multivariable analysis either due to few patients with *S. maltophilia* BSI having each characteristic thus limiting analysis or expected collinearity with other variables included within the model. While the StenoSCORE exhibited acceptable performance within our cohort, optimization of the score to include variables determined to be clinically important risk factors within our patient population significantly increased the performance of the StenoSCORE2. Multicenter validation of the StenoSCORE and StenoSCORE2 is warranted to assess external validity of our findings.

While awaiting final culture results, the use of clinical scoring tools may help identify patients at increased risk for a particular infection with the intent of decreasing time to appropriate antimicrobial therapy [20]. This is of increasing importance in the absence of rapid molecular diagnostic testing capabilities where organism identification can take over 24 hours to result and likely contributes to delayed appropriate antimicrobial therapy [21]. Risk scoring tools for identification of *S. maltophilia* infection may be particularly useful in decreasing time to active antimicrobial therapy as *S. maltophilia* is intrinsically resistant to anti-pseudomonal β-lactams and aminoglycosides which are used as empiric therapy in immunocompromised patients with suspected infection. While risk scores have been developed to differentiate *S. maltophilia* BSI from non-*S. maltophilia* Gram-negative or *P. aeruginosa* BSI, literature describing implementation of these scoring systems into clinical practice and their impact on patient outcomes is lacking [4, 12]. Our results demonstrated a significantly longer time to appropriate antimicrobial therapy in patients with *S. maltophilia* BSI compared with patients with non-*S. maltophilia* BSI (0.57 h vs. 45.16 h; *p* < 0001) as well as delayed appropriate antimicrobial therapy as an independent predictor of in-hospital mortality. Future studies assessing the feasibility of implementation of the StenoSCORE and/or StenoSCORE2 into clinical practice and their impact on time to appropriate antimicrobial therapy and mortality are warranted.

This study has limitations to consider. Firstly, although there were relatively few cases with *S. maltophilia* BSI within our cohort which may limit statistical power to assess risk factors for *S. maltophilia* BSI, the present study represents one of the largest studies systematically assessing *S. maltophilia* risk scoring tools. Similarly, we found overall similar performance of the StenoSCORE in an independent cohort providing external validation to this pragmatic risk tool. The StenoSCORE2 has yet to be externally validated outside of the current cohort. Lastly, the increased mortality observed in the *S. maltophilia* BSI group was likely impacted by multiple factors. In addition to delayed initiation of active antimicrobials in the *S. maltophilia* BSI group, unmeasured confounders such as malignancy disease status and BSI source were not assessed. Recently, the efficacy of antimicrobials traditionally used as first-line therapy for *S. maltophilia* infections under the previous clinical breakpoints has also been under scrutiny which may also contribute to worse outcomes in this patient population [22–24]. Future studies are needed to evaluate optimal empiric and susceptibility-directed therapy for *S. maltophilia* BSIs.

In conclusion, the StenoSCORE and StenoSCORE2 performed well in predicting *S. maltophilia* BSI in a cohort of patients with hematologic malignancy and/or HSCT and Gram-negative BSI. Risk factor derivation from a multicenter study would improve the accuracy and generalizability of these findings. To improve patient outcomes in *S. maltophilia* BSI, clinical studies evaluating the StenoSCORE and/or StenoSCORE2 implementation on time to effective therapy and clinical outcomes are warranted.

### Electronic supplementary material

Below is the link to the electronic supplementary material.

Supplementary Material 1

## Data Availability

Data supporting the results of this study are available from the corresponding author upon reasonable request.
