# Supplementary material for: Validation of a Stenotrophomonas maltophilia bloodstream infection prediction score in the hematologic malignancy population
Source: Ann Hematol. 2024 Mar 8;103(5):1745–52. doi: 10.1007/s00277-024-05686-z (PMC11009769; doi:10.1007/s00277-024-05686-z)
Supplement: Supplementary file 1 — Supplementary Material 1 [file 277_2024_5686_MOESM1_ESM.docx]

**Table S1. Non-*S. maltophilia* Gram-Negative Organism Data**

|  | **N = 534** |
| --- | --- |
| Isolated Organisms, N (%)  *Achromobacter sp.*  *Acinetobacter sp.*  *Aeromonas sp*  *Brevundimonas diminuta*  *Citrobacter freundii*  *Enterobacter aerogenes*  *Enterobacter cloacae*  *Escherichia coli*  *Klebsiella oxytoca*  *Klebsiella penumoniae*  *Klebsiella variicola*  *Morganella morganii*  *Pantoea agglomerans*  *Proteus mirabilis*  *Pseudomonas aeruginosa*  *Pseudomonas putida*  *Pseudomonas stutzeri*  *Serratia marcescens*  *Sphingomonas sp* | 10 (1.9)  4 (0.7)  4 (0.7)  1 (0.2)  7 (1.3)  7 (1.3)  57 (10.7)  206 (38.6)  20 (3.7)  131 (24.5)  26 (4.9)  2 (0.4)  1 (0.2)  1 (0.2)  114 (21.3)  2 (0.4)  1 (0.2)  6 (1.1)  3 (0.6) |
| Polymicrobial Gram-Negative BSI, N (%) | 63 (11.8) |
| Resistant Organisms, N (%)  Extended Spectrum Beta-Lactamase  Klebsiella Pneumoniae Carbapenemase  Carbapenem Resistant Enterobacterales | 35 (6.6)  5 (0.9)  24 (4.5) |

**Table S2. Factors Associated with *S. maltophilia* BSI using Multivariable Regression**

| **Variable** | **Β coefficient** | **Odds ratio (95% CI)** |
| --- | --- | --- |
| Acute myeloid leukemia or acute lymphoblastic leukemia | 0.68 | 1.98 (0.81-4.85) |
| Neutropenia ≥ 7 days | 1.41 | 4.10 (1.67-10.08) |
| Mucositis | 0.92 | 2.51 (1.08-5.79) |
| ICU admission within 12 hours of index culture | 0.67 | 1.94 (0.89-4.24) |
| Prior meropenem exposure ≥ 3 days | 1.53 | 4.63 (2.09-10.28) |
| Prior cefepime exposure ≥ 3 days | 0.94 | 2.55 (1.14-5.69) |
